# Deciphering key factors of active learning performance in biomolecular design

**DOI:** 10.1093/bioinformatics/btag248

**Published:** 2026-07-07

**Authors:** Yixuan Zhi, Qixiu Du, Han Yu, Lei Wei, Xiaowo Wang

**Affiliations:** Ministry of Education Key Laboratory of Bioinformatics, Center for Synthetic and Systems Biology, Beijing National Research Center for Information Science and Technology, Department of Automation, Tsinghua University, Beijing 100084, China; Ministry of Education Key Laboratory of Bioinformatics, Center for Synthetic and Systems Biology, Beijing National Research Center for Information Science and Technology, Department of Automation, Tsinghua University, Beijing 100084, China; Ministry of Education Key Laboratory of Bioinformatics, Center for Synthetic and Systems Biology, Beijing National Research Center for Information Science and Technology, Department of Automation, Tsinghua University, Beijing 100084, China; Ministry of Education Key Laboratory of Bioinformatics, Center for Synthetic and Systems Biology, Beijing National Research Center for Information Science and Technology, Department of Automation, Tsinghua University, Beijing 100084, China; Ministry of Education Key Laboratory of Bioinformatics, Center for Synthetic and Systems Biology, Beijing National Research Center for Information Science and Technology, Department of Automation, Tsinghua University, Beijing 100084, China; College of Artificial Intelligence, Tsinghua University, Beijing 100084, China

## Abstract

**Motivation:**

Employing machine learning (ML) to efficiently design biomolecules has become an emerging trend in genetic engineering. Active learning (AL) algorithms, as scalable approaches for ML-guided discovery, can automatically identify promising samples for function (i.e. fitness) optimization, and have therefore attracted growing interest across scientific domains. However, applying AL in genetic engineering presents several challenges. The regulatory patterns between sequence and fitness are highly complex, noisy, and sparse, making the existing evaluation of AL algorithm efficiency unreliable. Therefore, a comprehensive benchmark and thorough investigation into the key determinants of AL performance are urgently required to resolve these challenges.

**Results:**

We created a benchmark across multiple large-scale libraries of proteins and DNA regulatory sequences, evaluating uncertainty quantification (UQ) algorithms on metrics including calibration and accuracy, demonstrating the robustness and generality of ensemble-based algorithms. Moreover, we systematically assessed the efficiency of existing sampling strategies for fitness optimization. Our results show that no single sampling strategy is universally optimal across datasets, although greedy iterative strategies perform well in many practical scenarios. Finally, we evaluated the factors influencing optimization efficiency, and found that optimization efficiency is mainly determined by the choice of initial settings, distribution sparsity, and sequence similarity in high-fitness regions, rather than by the specific AL algorithm. Based on this, we proposed two quantifiable metrics to interpret the strategy performance and provide a practical reference for strategy selection. These findings offer valuable insights for the implementation of AL pipelines in biomolecular sequence design scenarios.

**Availability and implementation:**

The source code and supporting datasets used in this work are openly available on GitHub at https://github.com/WangLabTHU/biomolecule-al-decipher and have been archived on Zenodo at https://doi.org/10.5281/zenodo.19661002.

## 1 Introduction

Designing genetic sequences to enhance functionalities is a growing trend in precision medicine and bio-manufacturing. Machine learning (ML) revolutionizes this field by uncovering hidden regulatory patterns and facilitating precise *in silico* evolution, efficiently targeting functions beyond the reach of lengthy natural selection. ML has performed well in diverse genetic engineering implementations, including the designing of high-fitness (Wang *et al.* 2020), cell-type (Gosai *et al.* 2024, Agarwal *et al.* 2025)/tissue-specific (de Almeida *et al.* 2024), or species-specific (Zhang *et al.* 2025) regulatory sequences. Despite a series of successful applications, the high cost and time demands of biological experiments have led researchers to seek protocols with higher efficiency.

**Figure 1:**
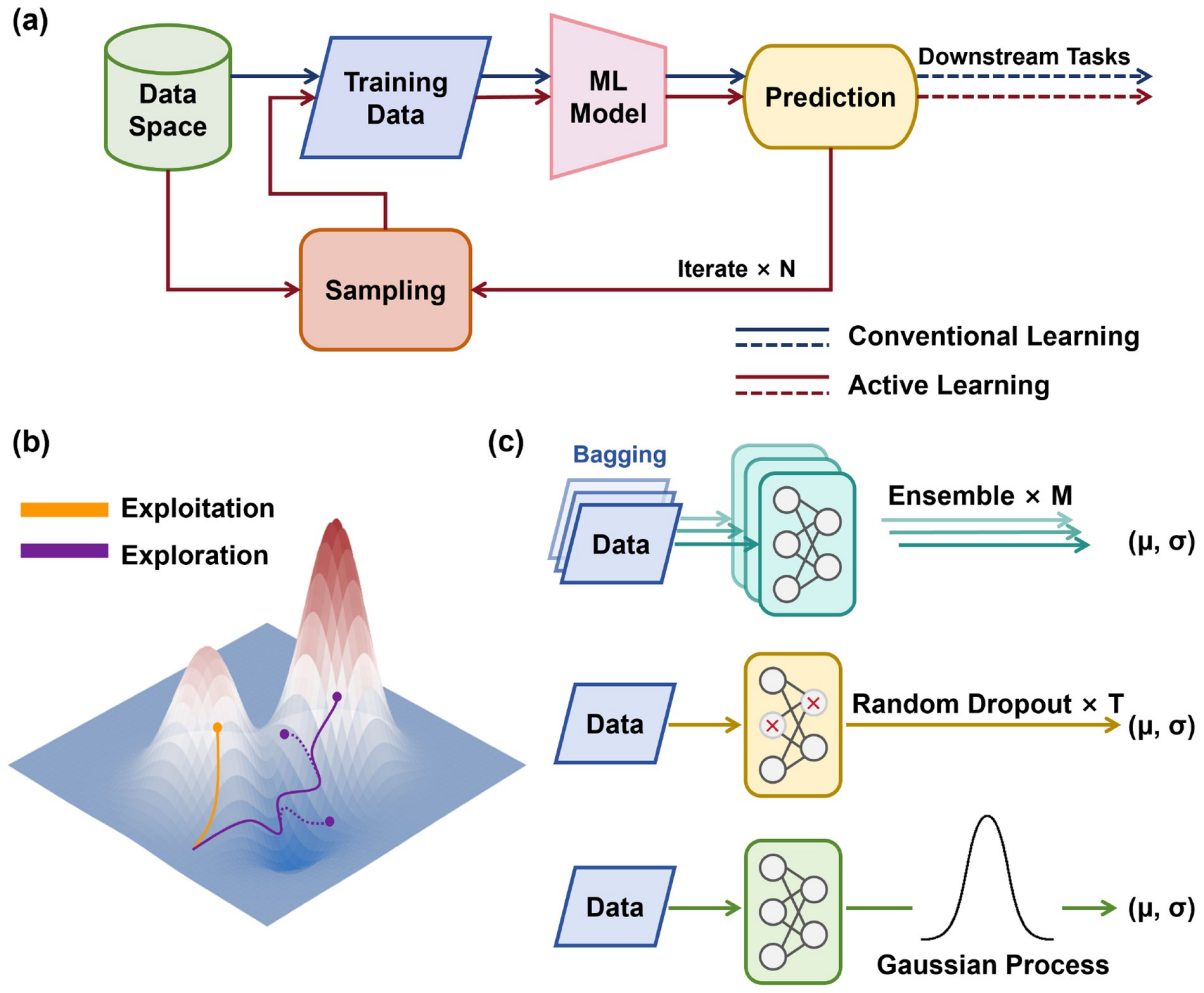
(a) Schematic illustration of acquisition paths for exploitative and exploratory sampling strategies in active learning on the data landscape. (b) Workflow comparison of active learning and conventional machine learning for biomolecular design. AL introduces a closed-loop sampling mechanism that iteratively updates training data, enabling more efficient model improvement compared to the one-way data flow of conventional learning. (c) Schematic of three UQ algorithms for AL: (1) ensemble-based algorithm, which trains M independent models on resampled data to quantify predicted uncertainty; (2) MC Dropout, which performs T forward passes with random neuron dropout to give uncertainty prediction; (3) DKL, which integrates NN with GP to characterize predictive distributions.

Active learning (AL) algorithms serve as a pivotal accelerator in the era of AI-driven scientific discovery. Initially proposed by Lewis (1995), this approach aims to achieve high accuracy with minimal labeling effort (Settles 2011). Specifically, AL algorithms leverage ML models to estimate predictions and uncertainties for candidates, and subsequently employ exploitative or exploratory acquisition functions (i.e. sampling strategy) to select promising samples, thereby iteratively improving model performance or optimizing functions compared with conventional learning (Fig. 1a). These algorithms have been widely adopted across diverse domains, including the discovery of novel stable crystals (Merchant *et al.* 2023), the optimization of chemical reaction conditions (Slattery *et al.* 2024), and the optimization of multicomponent alloys (Kusne *et al.* 2020). Notably, Frances Arnold’s laboratory has extensively utilized AL to guide protein-directed evolution, and identify optimal amino acid combinations (Rapp *et al.* 2024, Yang *et al.* 2025). Furthermore, Jiang *et al.* (2025) have recently utilized AL to enable the efficient design of diverse proteins, including antibodies, CRISPR nucleases, prime editors, and RNA polymerases.

Despite a series of successful implementations, applying AL to biomolecular sequence design remains challenging. The most prominent obstacle is the complexity of the sequence–fitness relationship, which renders uncertainty quantification (UQ) exceptionally difficult. For instance, designing regulatory DNA sequences involves complex, non-additive combinatorial mutational effects, where the space of candidate sequences expands exponentially with length (*L*), reaching $4^{L}$. Consequently, in practical scenarios, biological data are often locally biased and fail to fully capture the vast and intricate relationship between sequences and their fitness. These factors make accurate uncertainty modeling via machine learning exceptionally difficult, rendering traditional statistical approaches, such as Gaussian Processes (GPs), largely inapplicable (Yang *et al.* 2025).

Moreover, biomolecular sequence design is difficult due to the long-tailed distribution of fitness values. Most sequences are nonfunctional, while samples with the desired high fitness are rare, sparse, and widely dispersed within the sequence space. For instance, analysis of a simple 9 bp combinatorial library targeting critical regions of the dihydrofolate reductase *folA* gene shows that 93% of variants are nonfunctional (Papkou *et al.* 2023). Training datasets are typically composed of scarce functional sequences, resulting in a highly imbalanced fitness distribution. Given the high sparsity of this fitness landscape, it is questionable whether the sampling strategies employed in traditional AL algorithms remain effective, or if they can outperform simple exploitative iterations (Fig. 1b).

Despite numerous comparative studies on AL efficiency (Darmawan *et al.* 2025, Greenman *et al.* 2025, Yang *et al.* 2025), and our prior systematic review on AL in genetic engineering (Du *et al.* 2025), there remain some significant limitations. Current AL benchmarks primarily rely on simple amino acid combinatorial libraries (Wu *et al.* 2016, Chen *et al.* 2023, Johnston *et al.* 2024), where fitness landscapes are relatively smooth, and local neighborhoods around high-fitness samples are dense. However, benchmarks for complex scenarios like DNA regulatory sequence optimization are critically lacking, even though they reflect the challenges of practical scenarios involving massive search spaces, intricate fitness landscapes, and high sparsity. Current AL sampling algorithms often benchmark against random sampling (Rohr *et al.* 2020, Dave *et al.* 2022, Greenman *et al.* 2025). However, simple greedy iterations frequently outperform complex optimization strategies in real-world applications (Jiang *et al.* 2025). Therefore, comparing AL against exploitative strategies (e.g. greedy sampling) is more valuable for practical engineering. Also, despite extensive analyses yielding numerous uncertainty quantification and efficiency metrics (Guo *et al.* 2017), they remain scattered or primarily tailored for classification, necessitating systematic consolidation specifically for function optimization tasks.

In this study, we systematically curated a collection of standardized public DNA and protein datasets spanning diverse biological systems, including human, Saccharomyces cerevisiae (*S. cerevisiae*), and Escherichia coli (*E. coli*), etc. We assessed UQ capabilities using multiple metrics that cover different aspects of expected performance, including accuracy and calibration. Our results suggest that ensemble-based algorithms achieve optimal performance in biomolecule datasets. Moreover, evaluations of sampling strategies demonstrated that while no strategy is universally optimal, greedy iterations consistently deliver strong performance across many scenarios. Consequently, our results imply that optimization efficiency is fundamentally driven by the choice of initial settings and the distribution sparsity and sequence similarity in high-fitness regions. Specifically, with initial data dominated by low-fitness sequences, exploration–exploitation hybrid strategies (e.g. Thompson sampling) are more likely to reach the maximum fitness. Conversely, when high-fitness samples are dense or share high sequence similarity, the greedy strategy achieves the optimum. To interpret the AL performance of different strategies, we proposed two dataset-specific metrics (the High-fitness Sparsity Index, HSI; and the High-fitness Generalizability Index, HGI) which closely match our experiment results and provide an explanation for the observed performance. By mapping initial data onto our 14 datasets, these metrics provide a practical reference to proactively identify the most suitable sampling strategy. We believe these findings will support the implementation of AL-assisted genetic engineering and provide valuable insights to advance the frontiers of synthetic biology.

## 2 Materials and methods

### 2.1 Datasets

Building upon our previous collections and current research, we curated 14 publicly available datasets (Table 1). This collection includes four mutational datasets (three combinatorial libraries of amino acids and one of DNA) and 10 sequential datasets (four promoter datasets and six enhancer datasets), each consisting of biological sequences paired with functional measurements. Spanning diverse experimental techniques (MPRA and lentiMPRA) and sequence lengths ranging from 4 to 1000, these datasets exhibit varied distribution patterns of expression levels, thereby providing a diverse benchmark for evaluating active learning algorithms. We standardized all datasets into a unified CSV format containing aligned sequence–fitness pairs. We removed redundant barcode modifications, while sequences with incompatible lengths were removed to ensure consistency. Large datasets exceeding 100 000 samples were capped at 100 000 via random subsampling. Notably, fitness labels were used directly from the source studies without any preprocessing or transformation.

**Table 1:** Summary of datasets used in this study.

Dataset	Species*	Descriptions	Length	Total size	Dataset identifier	Reference
MPRALegNet_HepG2	hsa	enhancer	200	100 000	D1	Agarwal *et al.* (2025)
MPRALegNet_K562	hsa	enhancer	200	100 000	D2	Agarwal *et al.* (2025)
MPRALegNet_WTC11	hsa	enhancer	200	92 370	D3	Agarwal *et al.* (2025)
Malinois_HepG2	hsa	enhancer	200	100 000	D4	Gosai *et al.* (2024)
Malinois_K562	hsa	enhancer	200	100 000	D5	Gosai *et al.* (2024)
Malinois_SK-N-SH	hsa	enhancer	200	100 000	D6	Gosai *et al.* (2024)
Ecoli_Wang_2020	eco	promoter	50	11 884	D7	Thomason *et al.* (2015)
Ecoli_Wang_2023	eco	promoter	165	13 972	D8	Zhang *et al.* (2023)
Yeast_Aviv_2022	sce	promoter	80	100 000	D9	Vaishnav *et al.* (2022)
Yeast_Zelezniak_2022	sce	promoter	1000	4238	D10	Zrimec *et al.* (2022)
Gb1_Arnold_2024	**	protein	4	149 360	D11	Wu *et al.* (2016)
TrpB_Arnold_2024	eco	protein	4	159 128	D12	Johnston *et al.* (2024)
folA_Wagner_2023	eco	DNA	9	261 333	D13	Papkou *et al.* (2023)
CreiLOV_Tong_2023	cre	protein	15	165 428	D14	Chen *et al.* (2023)

Length: sequence length; total size: number of sequences.

* Species are symboled by abbreviations from KEGG: hsa, Homo sapiens; eco, Escherichia coli; sce, Saccharomyces cerevisiae; cre, Chlamydomonas reinhardtii.

** GB1 is a protein derived from *Streptococcus dysgalactiae*. Its function is defined by both the ability to fold and the ability to bind the IgG-Fc antibody. No KEGG tag is assigned.

### 2.2 Uncertainty quantification algorithms

We focus on three representative uncertainty quantification approaches that have been commonly employed in uncertainty modeling, including ensemble-based algorithms, Monte Carlo dropout (MC dropout), and deep kernel learning (DKL) (Gal and Ghahramani 2016, Wilson *et al.* 2016). These algorithms span different modeling assumptions and UQ mechanisms, and thus provide complementary perspectives on uncertainty in discrete biological sequence space. For consistency across algorithms, we set the ensemble size M=5 and the number of stochastic forward passes T=5 for MC dropout, in line with previous research (Yang *et al.* 2025) (Section 1.1, available as supplementary data at *Bioinformatics* online).

### 2.3 Sampling strategies

We focus on sampling strategies that utilize model predictions and uncertainties to select informative sequences, thereby optimizing the task that identify sequences with global maximum fitness. Specifically, we consider three representative strategies, including greedy sampling, Thompson sampling (TS), and upper confidence bound (UCB) sampling. For consistency in experimental design, the coefficient κ for UCB was set to 2, in line with previous research (Yang *et al.* 2025). The performance of these strategies was evaluated across all 14 datasets (Fig. 1c) (Section 1.2, available as supplementary data at *Bioinformatics* online).

### 2.4 Metrics for uncertainty evaluation

In order to systematically assess predicted uncertainty in AL biomolecular design scenarios, we employed metrics from prior studies to evaluate all UQ algorithms, including calibration curves (Yang *et al.* 2025) and the expected normalized calibration error (ENCE) (Pernot 2023). A uniform confidence grid and interval construction procedure were applied across all UQ algorithms and datasets to ensure the comparability of calibration curve results. For ENCE, a consistent binning strategy was implemented for all models and datasets (Section 1.3, available as supplementary data at *Bioinformatics* online).

### 2.5 Experimental framework for active learning in sequence–fitness prediction

#### 2.5.1 Sequence representation and model architecture

For all datasets, sequences were represented using one-hot encoding. Specifically, amino acid sequences were encoded into 4D vectors and protein sequences into 20D vectors. The models were trained to predict fitness from these encoded representations. For the ensemble-based algorithm and MC dropout, we employed two machine learning models: Multilayer perceptron (MLP) and Convolutional neural network (CNN) (Rumelhart *et al.* 1986, LeCun *et al.* 1998). For DKL, the model utilizes an MLP feature extractor with an architecture consistent with the aforementioned MLP, ensuring comparable representational capacity (Section 1.4.1, available as supplementary data at *Bioinformatics* online).

#### 2.5.2 Setup and assessment of UQ algorithms

We evaluated the reliability of three UQ algorithms across all 14 sequence-fitness datasets. For each combination of dataset and UQ algorithm, the sequences were randomly partitioned into training, validation, and test sets consisting of 3000, 500, and 500 samples, respectively. For consistent experimental design, all models were trained for 100 epochs with a batch size of 64 and an initial learning rate of $10^{-3}$. Key UQ algorithm parameters were fixed as follows: ensemble size M=5, MC dropout forward passes T=5 (dropout rate 0.2). Each dataset-UQ algorithm combination was repeated 5 times with different random seeds to account for stochasticity. After training, the reliability of UQ algorithms was assessed using calibration curves, ENCE, and PCC on the test set (Section 1.4.2, available as supplementary data at *Bioinformatics* online).

#### 2.5.3 Iterative active learning simulations

We simulated iterative active learning procedures on all 14 datasets to evaluate the optimization efficiency of three sampling strategies, based on uncertainty estimates from the ensemble-based algorithm (Sections 1.4.3 and 1.4.4, available as supplementary data at *Bioinformatics* online). To account for stochasticity, each simulation was repeated N=70 times with different random seeds, and the maximum observed fitness in the expanding training set was recorded at each round to measure optimization progress.

## 3 Results

### 3.1 Ensemble-based algorithm provides more reliable uncertainty quantification across sequence datasets

We evaluated three UQ algorithms (ensemble-based, MC dropout, and DKL) across all 14 sequence-fitness datasets. Specifically, the ensemble-based algorithm is a simple and efficient statistical method, while MC dropout serves as a representative algorithm for Bayesian neural networks and DKL acts as a standard algorithm of GP. Firstly, we assessed how well predicted uncertainties were calibrated using calibration curves. For a well-calibrated model, predictive intervals at a pre-defined confidence level should contain the corresponding proportion of actual fitness. In this case, the model’s calibration curve will closely follow the ideal diagonal. Thus, the miscalibration area is defined as the area between a calibration curve and the ideal diagonal and the area quantifies deviation from perfect calibration(Yang *et al.* 2025).

**Figure 2:**
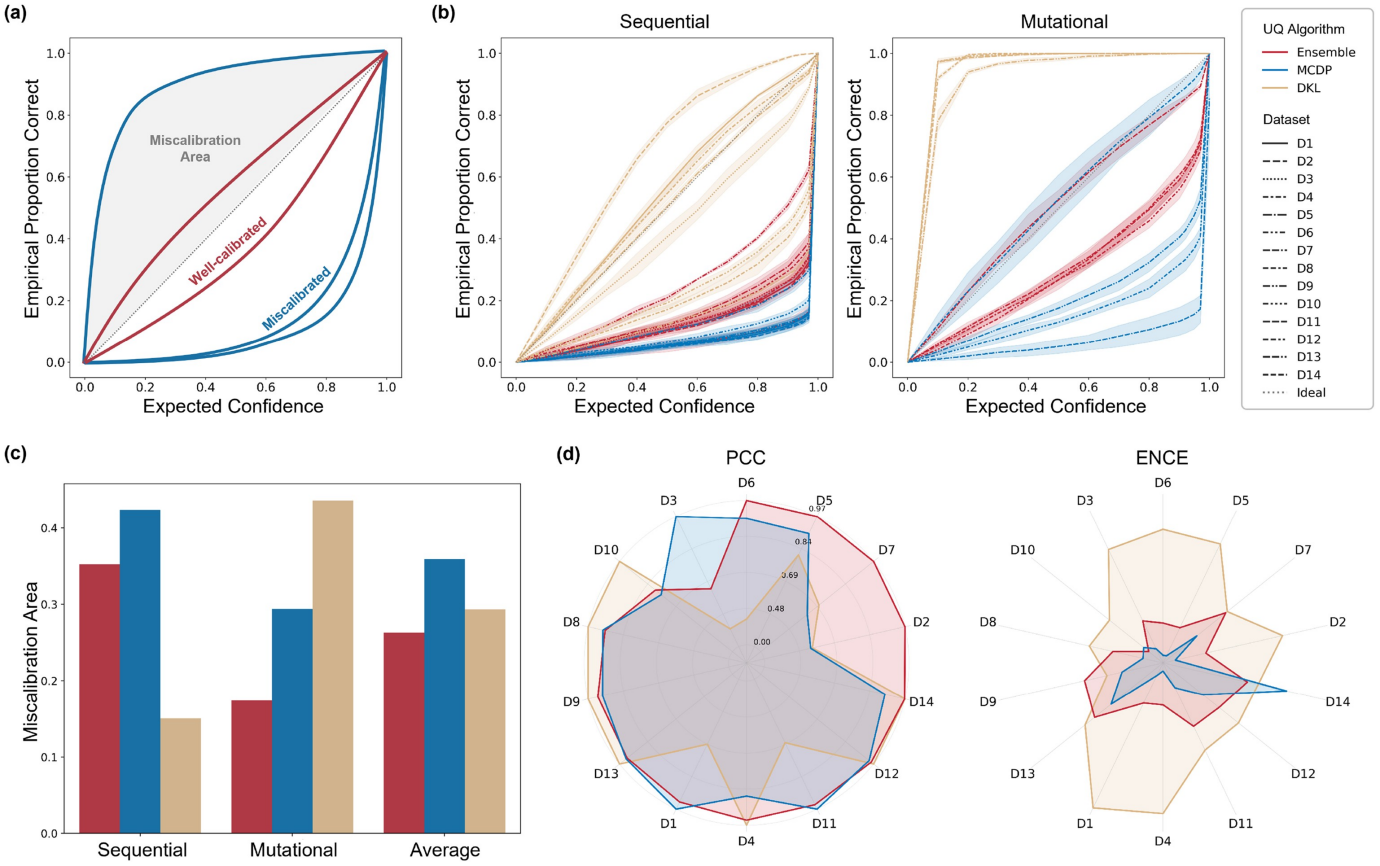
(a) Schematic illustration of calibration curves. For each curve, the *x*-axis represents the expected confidence (i.e. pre-defined confidence levels for predictive intervals), while the *y*-axis represents the empirical coverage (i.e. the actual proportion of true values falling within the intervals). Curves close to the ideal diagonal indicate well-calibrated models, and the area between a curve and the ideal diagonal is miscalibration area. (b) Calibration curves for 10 sequential datasets (D1–D10) using ensemble-based algorithm (Ensemble), MC dropout (MCDP), and DKL to quantify uncertainty; calibration curves for 4 mutational datasets (D11–D14) with the same experimental setups. (c) Average miscalibration area comparison across sequential, mutational, and their combined average for three UQ algorithms. (d) Radar plots on PCC (higher is better) and ENCE (negative log-transformed; higher is better) metrics across 14 datasets (D1–D14) for three UQ algorithms.

From these observations (Fig. 2a–c), we can summarize the calibration performance of the three methods as follows. DKL achieves superior calibration compared to the other two algorithms on several complex sequential datasets, especially those derived from human regulatory sequences. However, this advantage does not generalize across datasets, given that DKL yields substantially higher miscalibration area on mutational datasets. On sequential datasets, MC dropout consistently underperforms relative to others. On mutational datasets, MC dropout achieves comparable calibration quality to ensemble-based algorithms in some cases, but this behavior is not consistent across datasets. By comparison, ensemble-based algorithm gives the lowest average miscalibration area across both sequential and mutational datasets, indicating that it is the most well-calibrated algorithm.

Moreover, we examined the relationship between UQ performance and prediction accuracy, and assessed whether a model could simultaneously achieve reliable uncertainty estimation and strong predictive performance. To this end, we evaluated all algorithms using PCC as a measure of prediction accuracy and ENCE as a measure of uncertainty–error consistency. Higher PCC values indicate lower prediction error, while lower ENCE values indicate better alignment between predicted uncertainties and actual errors, together reflecting higher quality of uncertainty quantification. To facilitate intuitive visualization, we plotted −log(ENCE) in figures, where larger values denote superior calibration performance. A comparative assessment of all UQ algorithms across datasets based on PCC and −log(ENCE) is presented in Fig. 2c. From the perspective of consistency, DKL achieves the optimal ENCE performance on the majority of datasets, followed by ensemble-based algorithm, with MC dropout ranking last. However, on several sequential datasets where DKL yields the minimal ENCE values, it simultaneously produces the worst PCC among the three algorithms. Ensemble-based algorithm consistently demonstrates strong predictive accuracy. Across the 14 datasets, it yields a PCC among the top values in 12 cases, while maintaining competitive uncertainty–error consistency. MC dropout yields top PCC in 10 cases out of 14, yet exhibits bottom ENCE values in 10 cases, indicating its poor uncertainty–error consistency.

Taken as a whole, DKL appears to improve uncertainty–error consistency at the cost of substantial predictive accuracy. MC dropout, while maintaining relatively high predictive accuracy in many cases, fails to achieve consistent uncertainty–error alignment. Only ensemble-based algorithm combines strong predictive accuracy with competitive consistency. Importantly, uncertainty estimates are only meaningful when the underlying predictive accuracy is sufficiently high. Under these conditions, alignment between uncertainty and error can serve as a valid indicator of the well-calibrated uncertainty.

We further conducted a series of ablation studies to assess the robustness of UQ algorithm under changes in model architecture and hyperparameters (Sections 2.1 and 2.2, available as supplementary data at *Bioinformatics* online). For ensemble-based algorithm and MC dropout, we replaced the MLP base model with a three-layer CNN and re-evaluated miscalibration area. The resulting trends were highly consistent with those observed using MLPs, indicating that the conclusions were not sensitive to the choice of base model. Also, we varied the number of ensemble members and the number of stochastic forward passes for MC dropout (3, 5, and 10), and observed no notable difference. Similarly, varying the dropout rate in MC dropout (0.1, 0.2, 0.3, and 0.5) did not lead to qualitatively different outcomes. These results suggest that both ensemble-based algorithm and MC dropout exhibit strong robustness to architectural and hyperparameter variations.

In summary, these findings indicate that both ensemble-based algorithm and DKL have the potential to serve as effective UQ algorithm, depending on the dataset. However, from a practical perspective, ensemble-based algorithm emerges as the more reliable choice, as it is able to jointly maintain predictive accuracy and reliable calibration.

### 3.2 Behavior of active learning sampling strategies in sequence spaces

To characterize the behavior of different sampling strategies in biological sequence spaces, we conducted large-scale iterative active learning simulations using the ensemble-based predictive model. In total, we trained 8820 models, covering 14 datasets, three sample size configurations (initial training size and acquisition size) per dataset, and three sampling strategies (greedy sampling, TS, and UCB). Each dataset–sample size configuration–sampling strategy combination was repeated 70 times with different random seeds.

**Figure 3:**
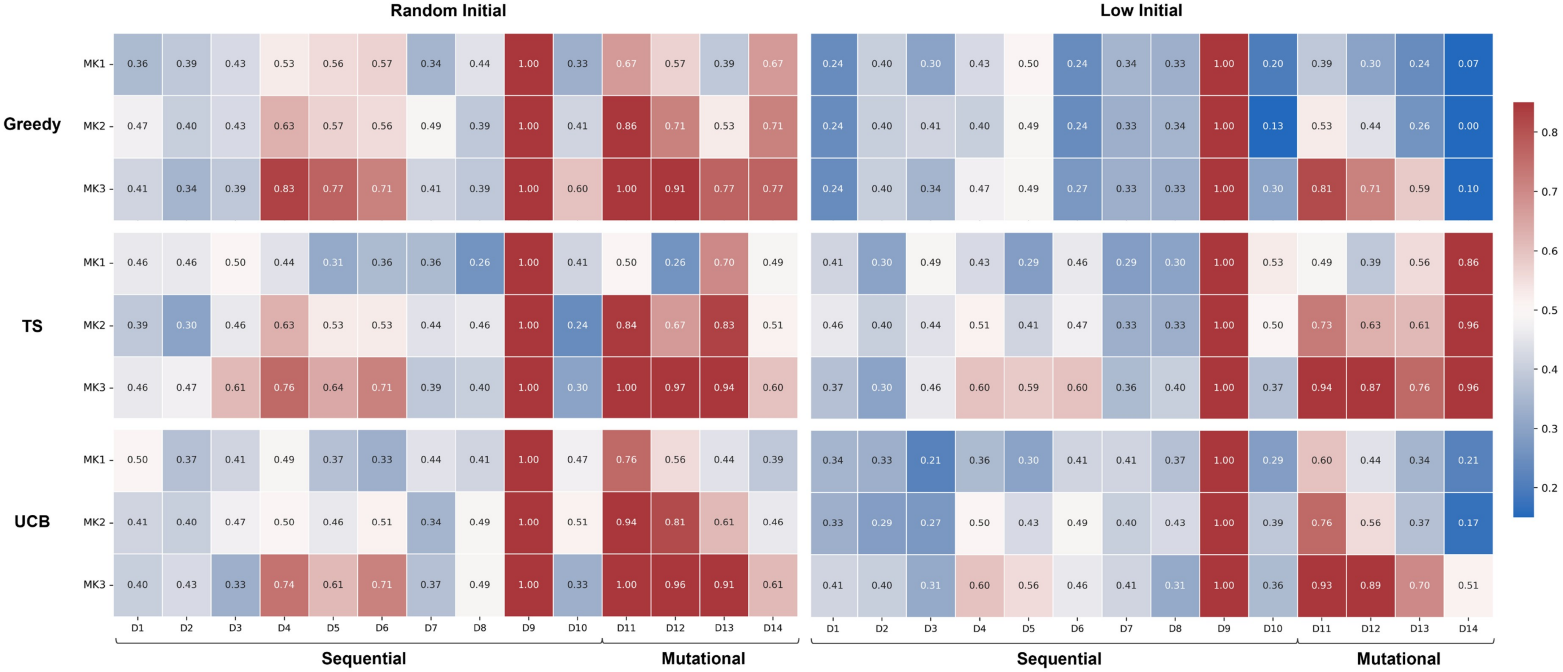
Heatmaps of the frequency of being the optimal strategy across 14 datasets (D1–D14), comparing three sampling strategies (greedy sampling, TS, UCB) under two initial settings (random initialization and low initialization) and three sample configurations (MK1, MK2, MK3). Redder colors indicate higher frequencies, while bluer colors indicate lower frequencies.

For each simulation, AL was initialized under a random initialization region by randomly sampling *M* sequences from the dataset to form the initial training set. After the final round, we recorded the maximum fitness observed and computed the frequency that each sampling strategy (greedy sampling, TS, or UCB) achieved optimal performance for each dataset and sample size configuration. These frequencies were summarized and visualized (Fig. 3) to provide a direct comparison of strategy efficacy across diverse datasets and configurations.

From these results, we gained a preliminary understanding of the relative suitability of exploitative strategies (e.g. greedy sampling) versus exploration–exploitation hybrid strategies (e.g. TS and UCB) across different datasets. Specifically, under all three sample size configurations, the greedy sampling strategy tended to sample sequences that achieve the highest fitness observed among the three sampling strategies for most sequential datasets (e.g. D4–D6, D10) and mutational datasets (e.g. D11, D12, D14).

However, for certain datasets including D1–D3 and D13, the greedy sampling strategy may underperform the exploration–exploitation hybrid strategies. We hypothesize that this performance gap could be associated with some dataset-specific factors, such as AL initial training set conditions, the sequence landscape, and the distribution of fitness values. We therefore carried out focused investigations to identify factors underlying the suboptimal performance of the greedy sampling strategy.

### 3.3 Exploration–exploitation hybrid strategies outperform greedy sampling under low-fitness initial settings

Building on the above experiments, we further trained another 8820 models under a low initialization region, where the initial training set was constructed by sampling *N* sequences exclusively from the bottom 20% of the fitness distribution. AL was then carried out for four rounds using the same three sampling strategies (greedy sampling, TS, and UCB). After the final round, we again recorded the maximum fitness observed by each model and computed the corresponding frequencies (Fig. 3).

Under this low-initial setting, the greedy sampling strategy immediately lost its advantage on several datasets where it previously had the optimal performance under random initialization, including datasets D4–D6, D12, and D14. In contrast, the exploration–exploitation hybrid strategies TS and UCB exhibited higher frequencies of optimal performance under this setting. Notably, for dataset D14, TS achieved nearly complete optimal performance among the other two strategies when initialized from low-fitness regions. For datasets D4–D6 and D11–D13, although TS and UCB also experienced some reduction in frequencies compared to random-initial setting, their performance degraded substantially less than that of the greedy sampling strategy, indicating greater robustness to low-fitness regions.

Overall, these results suggest that in biomolecular design scenarios where high fitness values are sought, AL initial settings starting from low-fitness regions can influence the effectiveness of sampling strategies. Under such settings, exploration-driven by uncertainty becomes increasingly important, favoring exploration–exploitation hybrid strategies over purely exploitative ones.

### 3.4 Steepness of rank-fitness curve challenges greedy sampling

Despite random initialization, we observed that the exploitation-driven greedy sampling strategy still performed poorly on several datasets. To further investigate the underlying causes of this behavior, we analyzed all datasets from the perspective of the sharpness of their fitness distributions.

**Figure 4:**
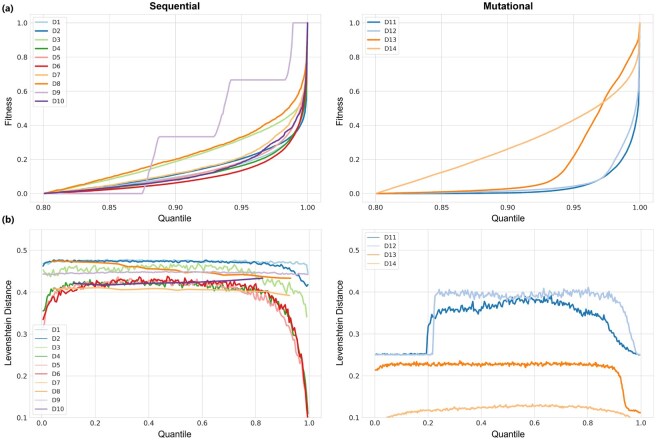
(a) Rank-fitness curves for 10 sequential datasets (D1–D10) and 4 mutational datasets (D10–D14), ranked by sequence fitness normalized within the 0.8–1.0 quantile range for cross-dataset comparison. Dataset D10 exhibits a stepped trend since the original fitness were sorted into uniformly sized expression bins. (b) Normalized Levenshtein distance (edit distance) curves for 10 sequential datasets (D1–D10) and 4 mutational datasets (D10–D14), computed across sliding windows of sequences and ranked by the fitness. Plateau in D11/D12 (0.0–0.2 quantile) due to few mutated sites, resulting in highly similar low-fitness sequences and nearly constant normalized edit distance (≈ 1).

For each of the 14 datasets, sequence data were ranked according to their fitness, and the corresponding values were normalized to enable comparison across datasets with different scales. These rank–fitness curves revealed that datasets on which greedy sampling underperformed exhibited markedly steeper increases in fitness in the upper quantiles (Fig. 4a).

In sequential datasets, some cases (e.g. D1, D2, D3) displayed an especially sharp rise within the top 20% quantile. Similarly, among the mutational datasets, dataset D13 showed substantially steeper value increases compared to the other three (D11, D12, D14), which exhibited a more gradual ascent. Notably, greedy sampling was precisely far more prone to lose its optimal performance on all these datasets. These steep rank–fitness curve profiles indicate a highly uneven distribution of fitness, where high-fitness sequences are sparse in a large region of the sequence space.

Such steepness in rank-fitness curves poses intrinsic challenges for exploitative sampling strategies. When high-fitness regions occupy only a narrow and sparsely sampled portion of the space, regulatory patterns learned by the model near these regions are more likely to be discontinuous or weakly informative. As a result, greedy sampling, which relies primarily on the predicted value, may struggle to consistently select the best acquisitions. In contrast, strategies based on a certain degree of exploration are better positioned to overcome these structural obstacles. In summary, these observations suggest that the steepness of the rank-fitness curves in upper quantiles, a profile that reflects the underlying sparsity in the distribution of fitness, constitutes an important dataset-specific factor influencing the effectiveness of different AL sampling strategies.

To quantify this key sparsity feature, we propose the High-fitness Sparsity Index (HSI). HSI is derived from the slope characteristics of the upper quantile of the rank-fitness curves, with higher values indicating sparser high-fitness distribution. Detailed computation steps for HSI are provided in Section 1.5.1, available as supplementary data at *Bioinformatics* online. This quantifiable metric enables objective characterization of the sparsity reflected by curve steepness, laying a foundation for comparative analysis across different datasets.

### 3.5 Rugged sequence landscapes constrain greedy sampling

Beyond the steepness, we identify sequence landscape ruggedness as another critical factor influencing the effectiveness of AL sampling strategies. We hypothesis that for some datasets fitness values exhibit local smoothness as the sequence edit distance (Levenshtein distance) decreases, enabling the model to extract stable and learnable patterns from a limited number of observations. In contrast, other landscapes are highly rugged, dominated by noise and substantial sequence diversity, where high-fitness sequences fail to form coherent local structures that can be reliably generalized.

To characterize the variations in sequence diversity across different sequence landscapes, we constructed sliding windows along the ranked fitness and computed the average normalized edit distance among sequences within each window (Fig. 4b). On certain datasets (e.g. D4, D5, D6), sequence distances progressively decreased as fitness increased, indicating increasing structural similarity among high-fitness sequences. Such contraction of the sequence landscape suggested the emergence of locally learnable neighborhoods, under which the greedy sampling could effectively ascend toward high-fitness regions. However, in the remaining datasets, the average edit distance within high-fitness windows remained consistently large at the top quantiles. This pattern implied that high-fitness sequences were structurally diverse and scattered, lacking shared motifs or local regulatory patterns. This phenomenon provided a direct explanation for the failure of greedy sampling in some datasets (e.g. D1–D3, D6–D8, and D10), where it struggled to identify a consistent ascent direction despite random initialization.

Taken together, these results demonstrate that the effectiveness of AL sampling strategies in biological sequence landscapes is strongly shaped by intrinsic landscape factors rather than by the sampling strategies alone. Exploitative sampling strategies as greedy sampling perform reliably only when high-fitness regions are sufficiently smooth, densely distributed, and when initial settings allow for the model to be within reach of such regions. In contrast, sparse high-fitness distributions and rugged sequence landscapes systematically undermine greedy exploitation, making exploratory strategies such as TS a more worthwhile choice. These observations highlight the necessity of aligning sampling strategies with dataset landscape features when deploying AL for biomolecular design.

To quantify the sequence similarity among high-fitness sequences, we propose the High-fitness Generalizability Index (HGI). HGI is calculated based on the neighborhood similarity of upper quantile sequences, with higher value indicating a smoother landscape with coherent high-fitness sequences. Detailed computation steps are provided in Section 1.5.2, available as supplementary data at *Bioinformatics* online. HGI quantitatively reflects the structural coherence of high-fitness sequences, enabling objective discrimination between rugged and smooth sequence landscapes.

### 3.6 Interpreting the performance of AL sampling strategies with HSI and HGI

In the preceding sections, we identified two critical dataset-specific landscape factors–steepness of the rank-fitness curves and ruggedness of the sequence landscape–that affect the performance of AL sampling strategies. To quantify these factors, we introduced HSI and HGI. Notably, our analysis reveals that the numerical values of HGI scale with the size of the dataset (Section 4, available as supplementary data at *Bioinformatics* online). Therefore, to ensure the comparability, we focus our analysis on datasets with similar sequence lengths and data scales: Group 1 (D1–D6) and Group 2 (D11, D12, and D14).

**Figure 5:**
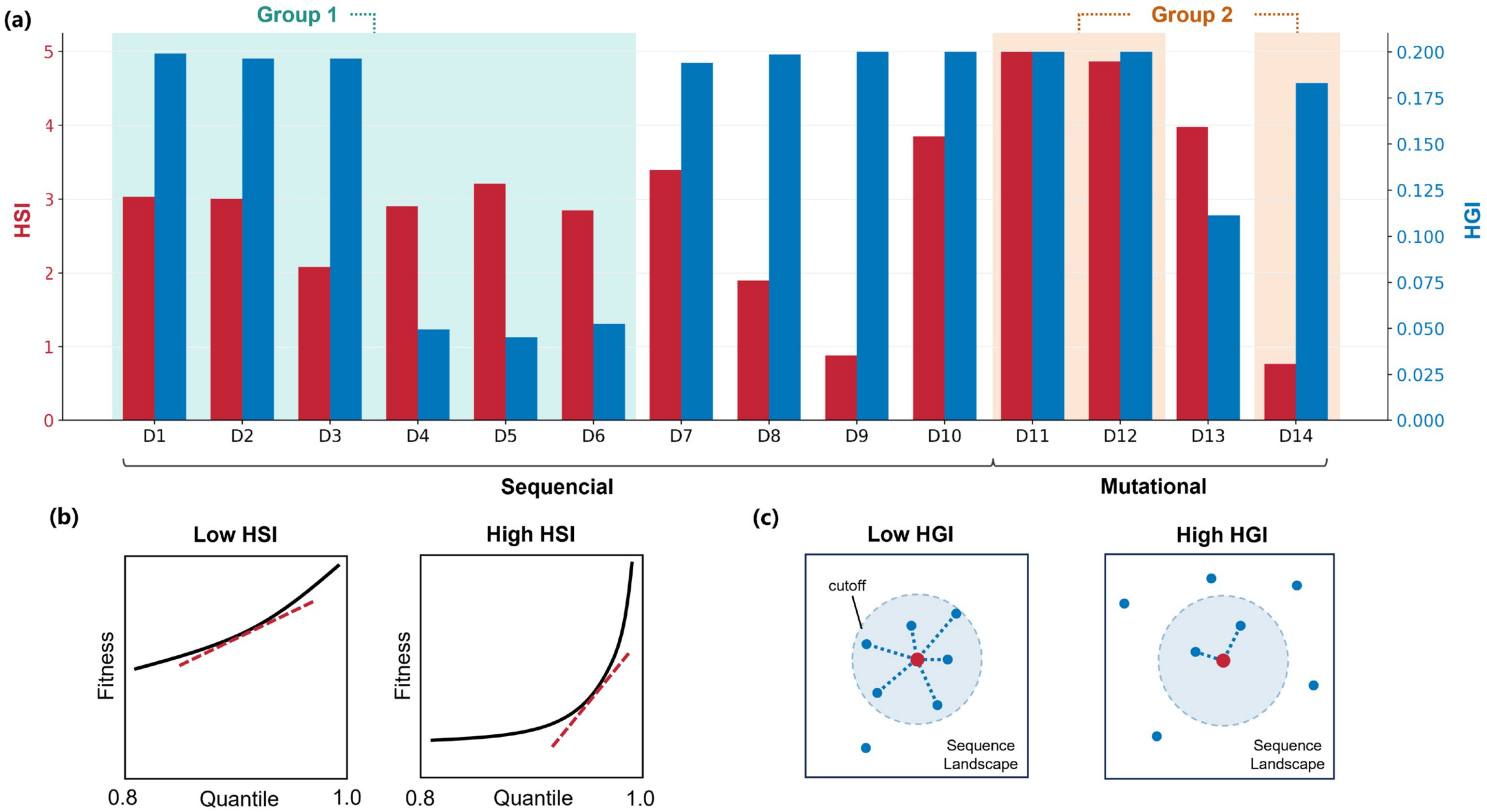
(a) The HSI (left bars in each pair; lower is better) and HGI (right bars in each pair; lower is better) of 14 datasets. The two shaded areas categorize Group 1 (D1–D6) and Group 2 (D11, D12, and D14), respectively. (b) Schematic illustration of HSI, demonstrating the steepness of upper quantile on rank-fitness curve and revealing the sparsity of the high-fitness region–with a lower HSI demonstrating a lower mean difference in the rank-fitness curve. (c) Schematic illustration of HGI, indicating the ruggedness of high-fitness sequence landscape and reflecting the degree of sequence similarity–with a lower HGI corresponding to a smaller average distance between a sequence and its neighboring sequences.

We computed HSI and HGI across all 14 datasets (Fig. 5a) and revealed an alignment between these metrics and the observed performance of AL sampling strategies. For instance, within Group 1, greedy performed optimally on datasets with lower HGI (D4–D6), where candidates are densely distributed. Furthermore, HSI exhibits a more dominant influence than HGI. As seen in Group 2, the low HSI of dataset D14 compensates for high HGI, indicating that sufficient high-fitness sequences are accessible to ensure optimal efficiency of greedy despite their structural diversity. Beyond these cases, remaining datasets exhibit high HSI and HGI, with modest greedy performance being surpassed by hybrid strategies. Collectively, as neither HSI nor HGI alone fully interpret the sampling strategy behaviors, their combination characterizes how strategy performances depend on specific biomolecular landscape factors.

Moreover, robustness analysis demonstrates that HSI and HGI remain consistent behavioral trends across subsets of varying scales randomly sampled from the original datasets (Section 4, available as supplementary data at *Bioinformatics* online). Consequently, the HSI and HGI of our 14 datasets provide a practical reference: by comparing the early-stage metrics of new data to our results for similar datasets (in terms of length and scale), researchers can proactively select the sampling strategy best suited for their AL pipeline.

## 4 Conclusion

In this work, we systematically evaluated the performance of UQ algorithms and AL sampling strategies across diverse datasets including DNA and protein, spanning multiple biological systems. Our analysis highlights that no single AL sampling strategies consistently outperforms others across all datasets. Instead, the efficiency of exploitation-driven and exploration-driven strategies is strongly influenced by AL initial settings and dataset-specific factors, particularly the sparsity and sequence similarity in high-fitness regions.

Beyond performance, our evaluation of UQ algorithms also reveals practical trade-offs. For instance, the ensemble-based algorithm offers interpretable uncertainty estimates, making it applicable across a wide range of base models. In contrast, DKL limits flexibility due to stronger structural assumptions as it treats upstream models as fixed embedding functions. These trade-offs suggest that practical constraints and theoretical appeal are both essential considerations for UQ selection in AL pipelines. Regarding sampling strategies, our findings underscore the challenge that sequence-fitness landscapes are not uniform, meaning that a strategy succeeding in one dataset may fail in another. This phenomenon potentially reflects that the absence of key motifs or functional substructures in the initial dataset can constrain the searchable space, whereas sampling strategies with stronger exploration capabilities may break these initial boundaries. Although our study focuses on sampling strategies, the construction of the initial dataset also serves as a critical factor for achieving robust AL success, which warrants further systematic investigation.

In conclusion, by combining a systematic evaluation of UQ algorithms and AL sampling strategies, with dataset-specific metrics HSI and HGI, this study provides both a robust benchmarking framework and conceptual insight for constructing AL biomolecular sequence design pipelines. We anticipate that these findings will inform the development of more effective AL workflows in synthetic biology and contribute to more efficient and targeted exploration of biomolecular landscapes.

## Supplementary Material

btag248_Supplementary_Data

## Data Availability

The source code and supporting datasets used in this work are openly available on GitHub at https://github.com/WangLabTHU/biomolecule-al-decipher.
