# Supplementary material for: Deciphering key factors of active learning performance in biomolecular design
Source: Bioinformatics. 2026 Jul 7;42(Suppl 1):btag248. doi: 10.1093/bioinformatics/btag248 (PMC13340224; doi:10.1093/bioinformatics/btag248)
Supplement: btag248_Supplementary_Data [file btag248_supplementary_data.pdf]

## Abstract

This document provides some experimental designs, experimental results, figures, and other materials not covered in the main text. The main focus of the supplement is to provide further analysis and to support the claims made in the main paper.

# Supplementary Materials: Deciphering Key Factors of Active Learning Performance in Biomolecular Design

# 1 Methods

This section provides the formalization of various algorithms, methods, and metrics that were not elaborated in detail in the Materials and methods section of the main text.

## 1.1 Uncertainty quantification algorithms

### 1.1.1 Ensemble-based algorithms

For a given sequence $x$, inference is performed by passing $x$ through all ensemble members $\{f_{\theta_1}, ..., f_{\theta_M}\}$. The predictive mean is defined as:

$$\mu(x) = \frac{1}{M} \sum_{m=1}^{M} f_{\theta_m}(x)$$

The predictive standard deviation (model uncertainty) is computed as:

$$\sigma(x) = \sqrt{\frac{1}{M} \sum_{m=1}^{M} (f_{\theta_m}(x) - \mu(x))^2}$$

The sequence prediction is taken as $\mu(x)$, and $\sigma(x)$ denotes the associated model uncertainty..

### 1.1.2 Monte Carlo dropout

Monte Carlo dropout model is trained on the full training dataset with standard dropout regularization; dropout remains enabled during inference, leading to stochastic forward passes for the same input sequence $x$.

Let $T$ denote the number of stochastic forward passes, with resulting predictions $\{f^{(1)}(x), ..., f^{(T)}(x)\}$. The predictive mean and predictive standard deviation are calculated as:

$$\mu(x) = \frac{1}{T} \sum_{t=1}^{T} f^{(t)}(x)$$

$$\sigma(x) = \sqrt{\frac{1}{T} \sum_{t=1}^{T} \left(f^{(t)}(x) - \mu(x)\right)^2}$$

The sequence prediction is taken as $\mu(x)$, and $\sigma(x)$ denotes the associated model uncertainty..

### 1.1.3 Deep kernel learning

Deep kernel learning integrates a sequence embedding feature extractor with Gaussian Process (GP) regression.

Let $\phi(x; \theta)$ denote the 3-layer MLP feature extractor (parameterized by $\theta$) mapping input $x$ to latent space, and $p(y|x; \theta, \Sigma)$ denote the GP distribution over target $y$ (conditioned on $\phi(x; \theta)$ and GP covariance parameters $\Sigma$). The predictive mean $\mu(x)$ and standard deviation $\sigma(x)$ are the mean and standard deviation of the GP predictive distribution:

$$\mu(x) = \mathbb{E}_{y \sim p(y|x;\theta,\Sigma)}[y]$$

$$\sigma(x) = \sqrt{\mathbb{V}_{y\sim p(y|x;\theta,\Sigma)}[y]} = \sqrt{\mathbb{E}_{y\sim p(y|x;\theta,\Sigma)}[(y-\mu(x))^2]}$$

The sequence prediction is taken as $\mu(x)$, and $\sigma(x)$ denotes the associated model uncertainty..

## 1.2 Sampling strategies

### 1.2.1 greedy sampling

By disregarding uncertainty, the greedy sampling strategy focuses exclusively on the current predicted optimum. This approach represents one of the simplest, yet quite effective, exploitative strategies and can be expressed as:

$$x^\star = \arg\max_x \hat{y}(x)$$

### 1.2.2 Thompson sampling

Thompson sampling is an approach designed to balance exploration and exploitation. Specifically, it constructs a posterior Gaussian distribution representing the current sample patterns by performing stochastic draws.

$$\tilde{y}(x) \sim \mathcal{N}(\hat{y}(x), \sigma^2(x))$$

After estimation, the candidates that maximize the posterior distributions will be selected.

$$x^\star = \arg\max_x \tilde{y}(x)$$

### 1.2.3 UCB sampling

UCB sampling selects candidates by linearly combining predicted mean and uncertainty:

$$x^\star = \arg\max_x(\hat{y}(x) + \kappa\sigma(x))$$

UCB balances exploration and exploitation trade-off controlled by coefficient $\kappa$.

## 1.3 Metrics for uncertainty evaluation

### 1.3.1 Calibration curves

For an input sequence $x_i$, let the model output a prediction $\hat{y}_i$ and uncertainty $\sigma_i$. For a desired confidence level $\alpha$, the predictive interval is constructed:

$$P(y_i \in [\hat{y}_i - z_{\alpha/2}\sigma_i, \hat{y}_i + z_{\alpha/2}\sigma_i]) \approx \alpha$$

where $z_\alpha = \sqrt{2}\,\mathrm{erfinv}(\alpha)$ is the scaling factor corresponding to the confidence level.

The empirical coverage is then computed as the fraction of true labels $y_i$ that fall within their respective predictive intervals:

$$\mathrm{Coverage}(\alpha) = \frac{1}{N}\sum_{i=1}^{N} \mathbf{1}\{y_i \in [\hat{y}_i - z_\alpha \sigma_i, \hat{y}_i + z_\alpha \sigma_i]\}$$

Plotting coverage as a function of nominal confidence $\alpha$ yields the calibration curve. The calibration curve plots these expected confidence levels on the x-axis against the empirical fraction of true labels that actually fall within the predicted intervals on the y-axis.

### 1.3.2 Expected Normalized Calibration Error

Partition the data into $B$ bins $b_1, \ldots, b_B$ according to the magnitude of predictive uncertainty $\sigma_i$, and denote by $|b_j|$ the number of points in bin $j$. Compute the root-mean-square uncertainty and root-mean-square error in each bin:

$$\mathrm{RMU}j = \sqrt{\frac{1}{|b_j|} \sum i \in b_j \sigma_i^2}$$

$$\mathrm{RME}j = \sqrt{\frac{1}{|b_j|} \sum i \in b_j e_i^2}$$

The ENCE is then defined as the average normalized discrepancy between predicted uncertainty and observed error across bins:

$$\mathrm{ENCE} = \sum_{j=1}^{B} \frac{|b_j|}{N} \cdot \frac{|\mathrm{RMU}_j - \mathrm{RME}_j|}{\mathrm{RMU}_j},$$

where $N$ is the total number of sequences.

## 1.4 Experimental setups

### 1.4.1 Model architecture

**Multilayer perceptron (MLP):** We employed a three-layer MLP as one of the base predictors in ensemble-based algorithm and MC dropout. It was implemented in Pytorch and using ReLU activations.

**Convolutional neural network (CNN):** We employed a three-layer CNN as another base predictors in ensemble-based algorithm and MC dropout. It was also implemented in Pytorch, using ReLU for activations and adaptive average pooling for handling variable-length inputs.

**Deep Kernel Learning model:** We used an MLP with the same architecture as in the ensemble-based algorithm and MC dropout as the feature extractor in DKL, after which a GP model was implemented using GpyTorch. This ensured comparable representational capacity across all UQ algorithms.

### 1.4.2 Setup of UQ algorithms

**Ensemble-based algorithm and MC dropout:** For each dataset, an MLP model was trained by minimizing the mean squared error loss using the Adam optimizer with an initial learning rate of $10^{-3}$. The training process was performed for 100 epochs with a batch size of 64. For ensemble-based algorithm, the number of ensemble members was fixed to 5. For MC dropout, the number of stochastic forward passes was fixed to 5 and the dropout rate was set to 0.2. Each dataset–UQ algorithm combination was repeated five times with different random seeds to account for stochasticity in model initialization and data sampling.

**DKL:** For each dataset, a DKL model consisting of an MLP feature extractor and a GP layer was trained. The model parameters, including both the MLP and GP hyperparameters, were optimized using the Adam optimizer with an initial learning rate of $10^{-3}$. The exact marginal log-likelihood under a Gaussian likelihood was maximized to learn the GP parameters using GPyTorch. The training process was performed for 100 epochs. The same five repetitions with different random seeds were applied to ensure fair comparison across all dataset–UQ algorithm combinations.

### 1.4.3 Setup of sample size

TableS1 presents the parameter configurations of initial training size (M) and acquisition size (K) for active learning across 14 datasets. For all three configurations (MK1, MK2, MK3), the value of M is consistent with K, and the specific numerical values are shown in the table.

Table S1: Sample Size Configuration for Different Datasets

| Dataset | Dataset Identifier | MK1 | MK2 | MK3 |
|---|---|---|---|---|
| MPRALegNet_HepG2 | D1 | 96 | 192 | 384 |
| MPRALegNet_K562 | D2 | 96 | 192 | 384 |
| MPRALegNet_WTC11 | D3 | 96 | 192 | 384 |
| Malinois_HepG2 | D4 | 96 | 192 | 384 |
| Malinois_K562 | D5 | 96 | 192 | 384 |
| Malinois_SKNSH | D6 | 96 | 192 | 384 |
| Ecoli_Wang_2020 | D7 | 10 | 20 | 40 |
| Ecoli_Wang_2023 | D8 | 10 | 20 | 40 |
| Yeast_Aviv_2022 | D9 | 480 | 960 | 1920 |
| Yeast_Zelezniak_2022 | D10 | 10 | 20 | 40 |
| Gb1_Arnold_2024 | D11 | 96 | 192 | 384 |
| TrpB_Arnold_2024 | D12 | 96 | 192 | 384 |
| folA_Wagner_2023 | D13 | 96 | 192 | 384 |
| CreiLOV_Tong_2023 | D14 | 96 | 192 | 384 |

### 1.4.4 Iterative active learning simulations

The active learning simulations were conducted using the ensemble-based model, with the MLP architecture and training setup kept identical to those described above. For each simulation replicate, the model was iteratively retrained on an expanding training set, and new sequences were sampled at each round based on the specified sampling strategy.

**Algorithm S1** Iterative Active Learning Simulation

**Require:** Dataset $\mathcal{D}$, initial training size $M$, acquisition size $K$, number of rounds $R = 4$, sampling strategy $\mathcal{S} \in \{\text{greedy sampling}, \text{TS}, \text{UCB}\}$. Both $M$ and $K$ were determined based on the total size of each dataset.

Randomly sample an initial training set $\mathcal{D}_{\text{train}}^{(0)}$ of size $M$
Initialize unlabeled pool $\mathcal{D}_{\text{pool}} = \mathcal{D} \setminus \mathcal{D}_{\text{train}}^{(0)}$
**for** $r = 1$ to $R$ **do**
  Train predictive model on $\mathcal{D}_{\text{train}}^{(r-1)}$
  Compute predictions and uncertainty estimates for all sequences in $\mathcal{D}_{\text{pool}}$
  Select $K$ sequences $\mathcal{D}_{\text{acq}}^{(r)}$ from $\mathcal{D}_{\text{pool}}$ using strategy $\mathcal{S}$
  Update training set $\mathcal{D}_{\text{train}}^{(r)} = \mathcal{D}_{\text{train}}^{(r-1)} \cup \mathcal{D}_{\text{acq}}^{(r)}$
  Update pool $\mathcal{D}_{\text{pool}} = \mathcal{D}_{\text{pool}} \setminus \mathcal{D}_{\text{acq}}^{(r)}$
  Record the maximum observed functional value in $\mathcal{D}_{\text{train}}^{(r)}$
**end for**

## 1.5 HSI (High-fitness Sparsity Index) and HGI (High-fitness Generalizability Index)

### 1.5.1 HSI

For the normalized fitness values $y_{\text{norm}}$ (scaled to $[0, 1]$ by min-max normalization), extract the quantile values of $y_{\text{norm}}$ over the quantile range $[0.8, 1.0]$ with 100 evenly spaced bins. Compute the slopes from the finite differences between adjacent quantile points, and HSI is defined as the mean of these slopes:

$$\text{HSI} = \frac{1}{99} \sum_{i=1}^{99} \frac{y_{\text{norm},q_i} - y_{\text{norm},q_{i-1}}}{q_i - q_{i-1}}$$

where $q_i$ denotes the $i$-th quantile point in the range $[0.8, 1.0]$.

### 1.5.2 HGI

For the selected top 5% sequences ranked by fitness, compute the pairwise Levenshtein distance matrix, then define the neighborhood radius $\epsilon$ as 20% of the sequence length. For each sequence in the top 5% set, identify its neighboring sequences within the distance $\epsilon$; if the number of neighbors is less than 1, assign a penalty value of 0.2 to this sequence, otherwise calculate the normalized average neighbor distance by dividing the average distance by the sequence length. HGI is finally defined as the mean of these values across all sequences in the top 5% set, formulated as:

$$\text{HGI} = \frac{1}{N_{\text{top5\%}}} \sum_{i=1}^{N_{\text{top5\%}}} h_i$$

where $h_i$ denotes the assigned value (either 0.2 or the normalized average neighbor distance) for the $i$-th sequence in the top 5% set, and $N_{\text{top5\%}}$ is the total number of sequences in this set.

# 2 Results on the ablation of calibration curves

## 2.1 Ablation of calibration curves for uncertainty quantification

Ablation experiments were performed on three variables: model number (ensemble size or MC dropout forward passes), model type (CNN/MLP), and dropout rate. Figure S1 shows calibration curves with ensemble-based and MC dropout algorithms of different setups, arranged in a $6 \times 4$ grid (the curves of DKL algorithm are consistent with those in the main text). The 6 rows correspond to combinations of model size (or forward passes) (3, 5, 10) and architectures (CNN, MLP), ordered top to bottom as 3-CNN, 3-MLP, 5-CNN, 5-MLP, 10-CNN, 10-MLP. The 4 columns represent dropout rates (0.1, 0.2, 0.3, 0.5) from left to right.

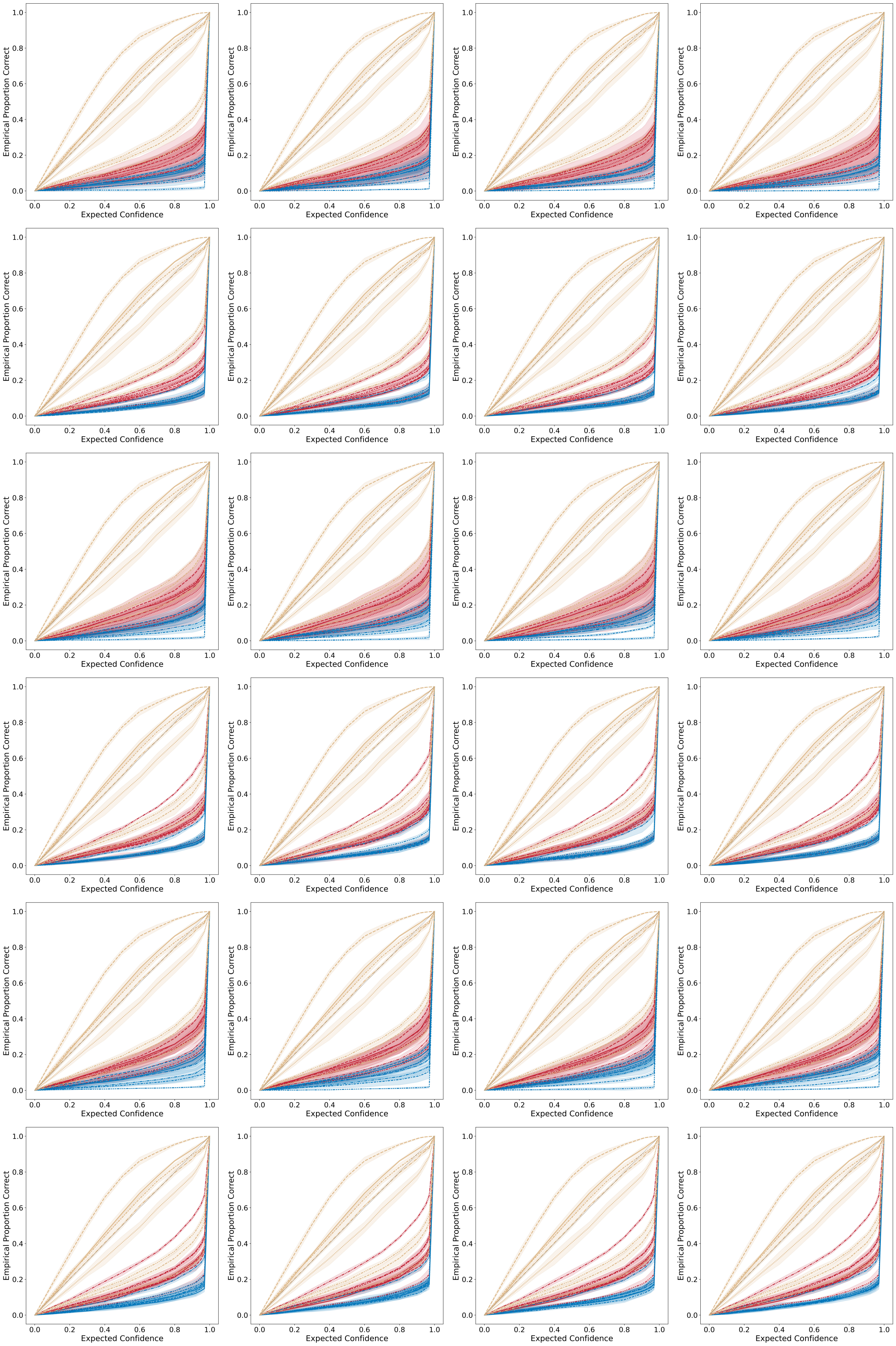


Figure S1: Calibration Curves of different setups

### 2.2 Assessment of ablation of calibration curves for uncertainty quantification

Figure S2 presents relative miscalibration areas for different UQ setups (model number, model type, and dropout rate). For each subplot, a single variable was varied while the other two were kept constant. Heatmap values denote relative miscalibration areas, normalized against the mean area of the varied variable across all conditions for the same dataset. The upper row displays ensemble-based results (left: model number; right: model type), and the lower row displays MC dropout results (left to right: dropout rate, model number, model type).

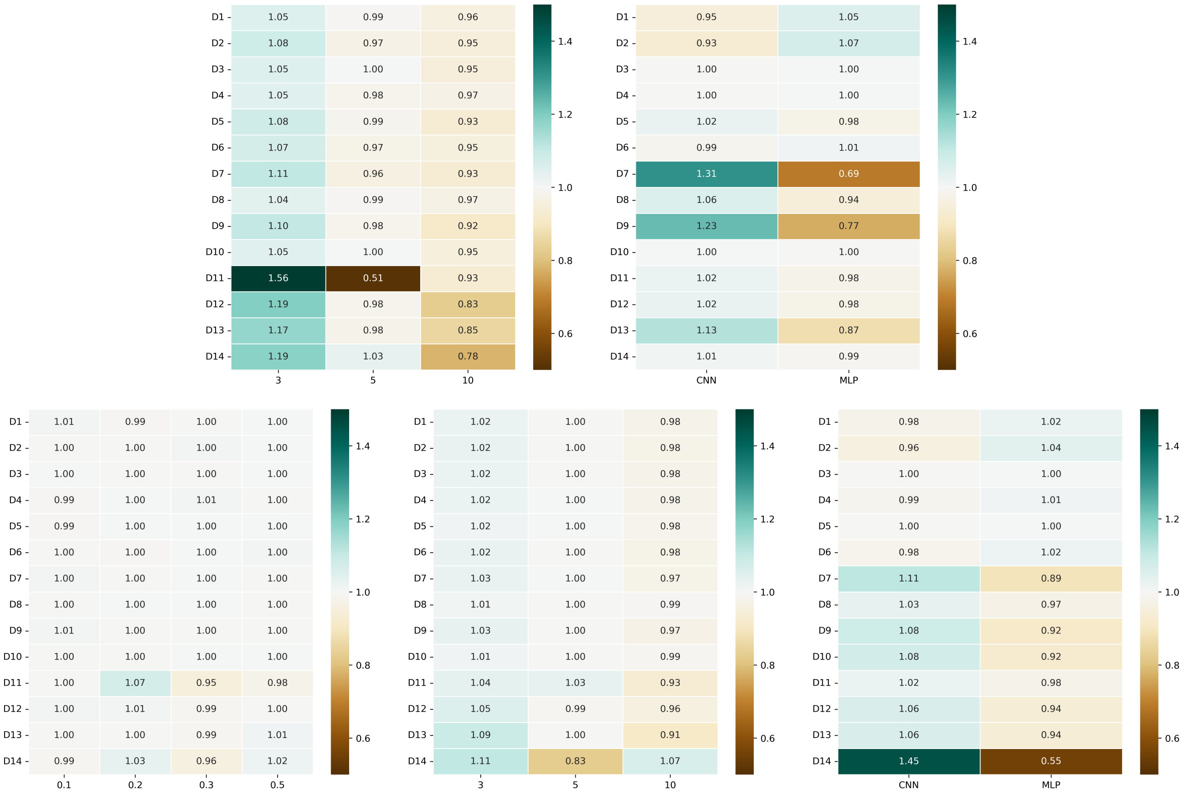


Figure S2: Relative miscalibration area of different setups

## 3 Comparison with random sampling and uncertainty-driven sampling strategies

To evaluate the specific contributions of exploration and exploitation to sampling performance, we extended our analysis to include two additional sampling scenarios: (i) uninformed random sampling and (ii) uncertainty-driven sampling.

### 3.1 Random sampling

As an uninformed control, random sampling selects candidates from the search space $\mathcal{X}$ with uniform probability, without utilizing any model-based predictions or uncertainty estimates:

$$x^\star \sim \mathcal{U}(\mathcal{X})$$

### 3.2 Uncertainty-driven sampling

This strategy purely focuses on exploration by prioritizing regions of the sequence space where the model's predictions are least certain. It disregards the predicted fitness values and selects candidates that maximize the predicted variance:

$$x^\star = \arg\max_x \sigma(x)$$

### 3.3 Experimental setups

We performed these evaluations on four representative datasets: D7 (Ecoli_Wang_2020), D11 (TrpB_Arnold_2024), D12 (folA_Wagner_2023), and D13 (CreiLOV_Tong_2023). The AL simulations followed the identical configurations described in the main text:

- **Initialization settings:** Random initialization and low-fitness initialization.
- **Sample size configurations ($M, K$):** D7: (10, 10), (20, 20), (40, 40); D11–D13: (96, 96), (192, 192), (384, 384).
- **Sampling strategies:** Random sampling and uncertainty-driven sampling, compared against Greedy, TS, and UCB.

Each experiment was repeated 70 times with different random seeds (consistent with the main text) to ensure robust performance trajectories.

### 3.4 Results

Across both random and low-fitness initialization settings (Figure S3), these two additional strategies consistently underperform the primary methods (Greedy, TS, and UCB). Notably, in datasets D11 and D12, the frequency with which random sampling identifies candidates with the highest fitness value is nearly zero, further validating the underlying efficacy of model-informed sampling strategies used in the work. While the uncertainty-driven strategy shows marginal improvements over random sampling, it fails to outperform the three primary strategies in the majority of experimental trials. These results reinforce our original findings that balancing exploration and exploitation is essential, especially in sparse biomolecular landscapes.

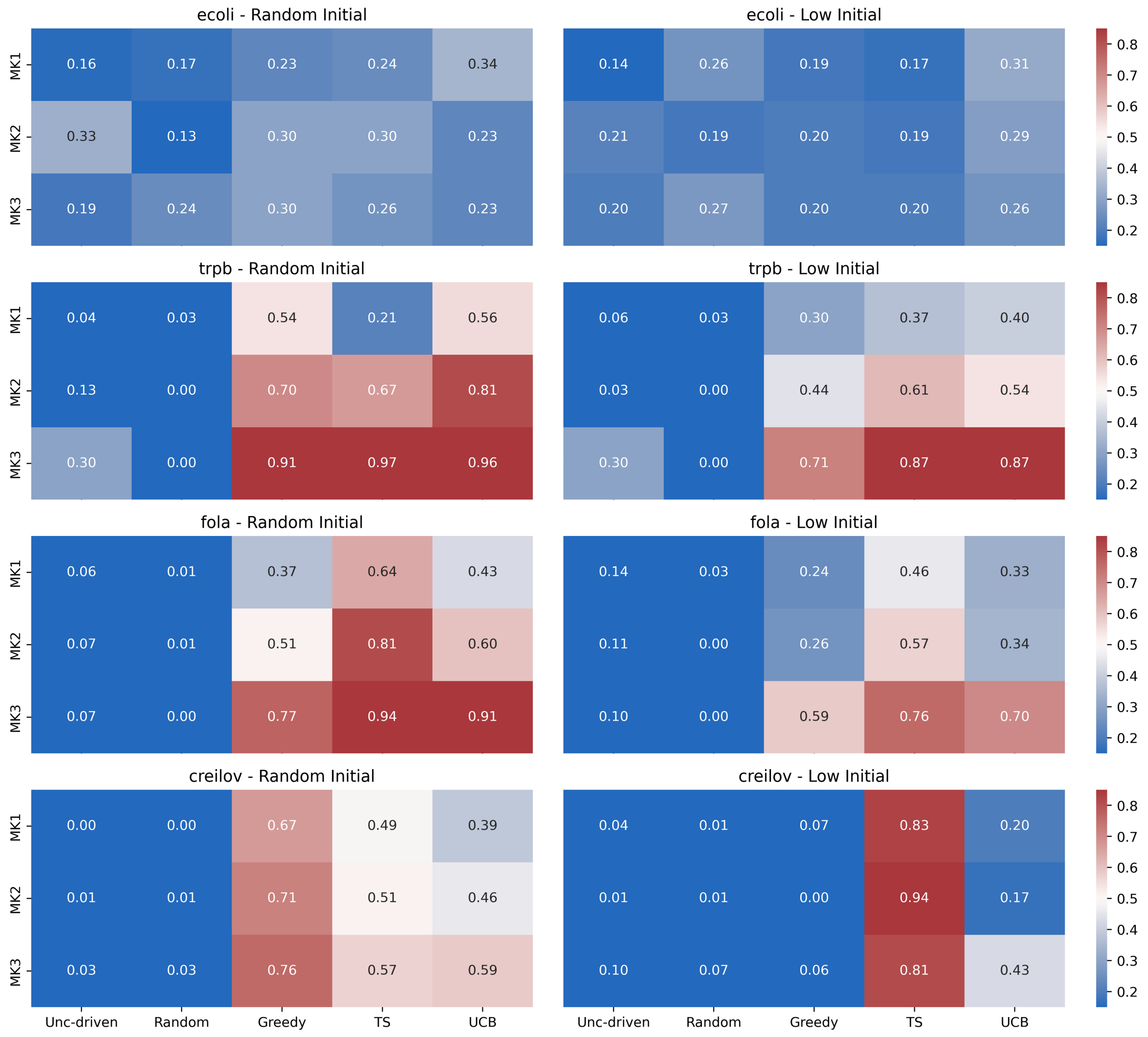


Figure S3: Frequency of achieving optimal performance across four representative datasets: ecoli (D7), trpb (D11), fola (D12), and creilov (D13), with three sample size configurations and five sampling strategies (Unc-driven stands for uncertainty-driven sampling; Random stands for random sampling).

# 4 Robustness analysis of HSI and HGI on data subsets

## 4.1 Experimental setups

To explore whether HSI and HGI can provide insights during the early stages of an AL pipeline, we evaluated their stability by extracting random subsets from the original datasets. For HSI, we sampled at the ratios of 1%, 5%, 10%, 20%, and 40% across 14 datasets, yielding 70 subsets in total. For HGI, as the numerical values scale with the sample size, we analyzed Group 1 (D1–D6) and Group 2 (D11, D12, and D14) separately while maintaining comparability within each group. The HSI and HGI calculated based on these randomly sampled subsets are summarized in Figure S4.

## 4.2 Results

For HSI, our results indicate that across varying sampling ratios, the metric remains stable and can reflect the high-fitness distribution sparsity of the full dataset even at small scales. This consistency suggests that HSI is a robust indicator of landscape sparsity at the early stage of an AL pipeline, ensuring that the profiling derived from initial libraries remains representative of the entire dataset.

For HGI, we observed that the numerical values decrease as the subset scale increases. Within each group, the relative HGI rankings and behavioral trends among different datasets remain consistent across all subset scales. These rankings suggest that HGI is comparable across datasets with similar sequence lengths, scales, and types (e.g., within Group 1 or Group 2). Furthermore, these same trends show that within comparable groups, HGI can reliably reflect the ruggedness of the entire dataset from the start, making it possible to use HGI for sampling strategy selection during the early stages of an AL pipeline.

Consequently, our results provide a practical reference for new datasets. For a new library, researchers can first identify datasets with similar data modalities among our 14 datasets and then calculate HSI and HGI from a randomly sampled subset. By comparing these values against our reference, it helps identify the most suitable sampling strategy for their own AL pipeline.

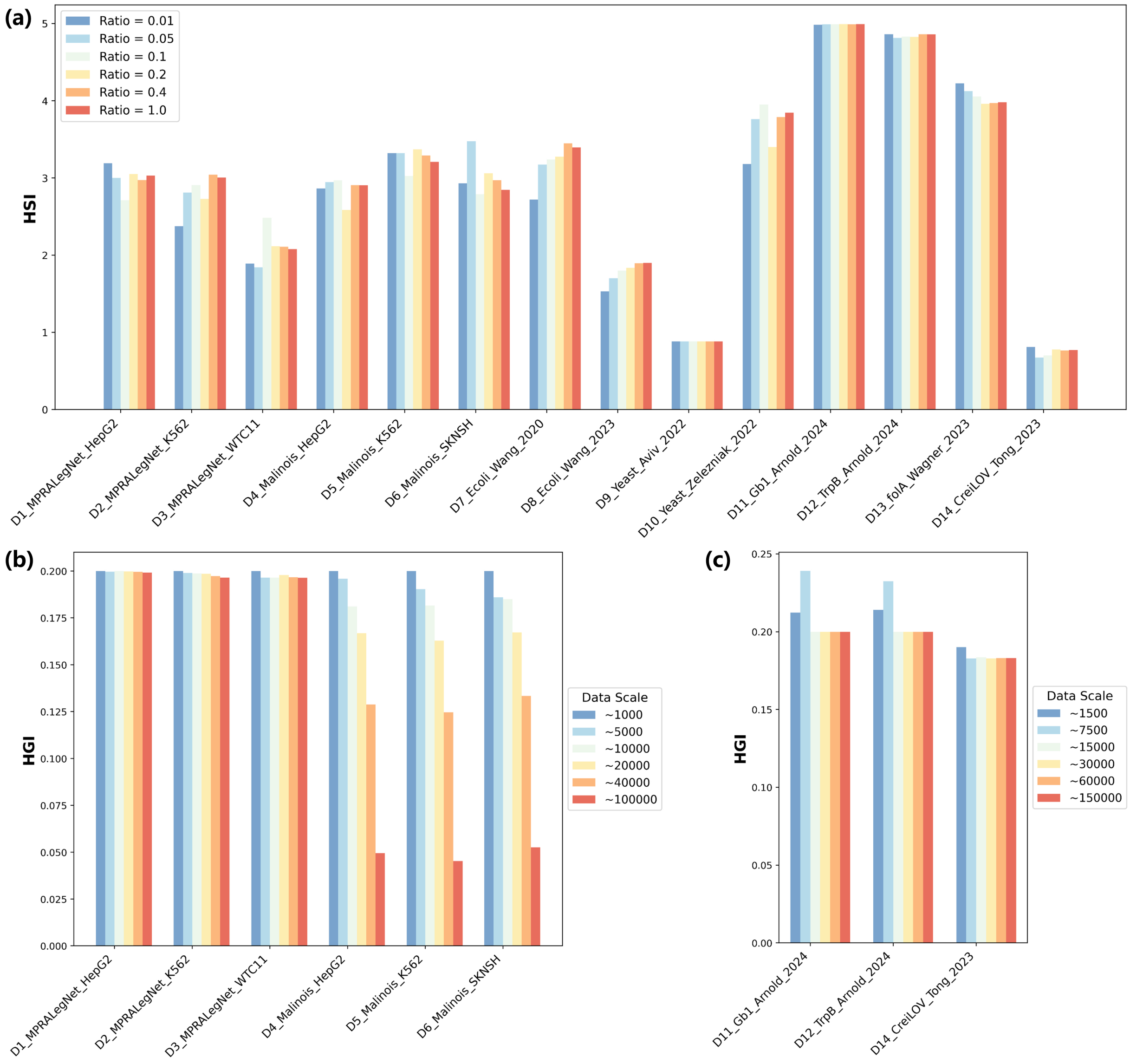


Figure S4: Robustness of landscape metrics (HSI and HGI) on sampled data subsets. (a) HSI for all 14 datasets, where grouped bars at each dataset represent different sampling ratios. (b) HGI transitions for Group 1 (D1–D6) across subset scales from 1,000 to 100,000. (c) HGI transitions for Group 2 (D11, D12, and D14) across subset scales from 1,500 to 150,000.
